# Can a national storage obligation for medicines prevent shortages? Evidence from the Finnish experience

**DOI:** 10.1016/j.rcsop.2025.100637

**Published:** 2025-07-22

**Authors:** Reko Ravela, Timi Aaltonen, Marja Airaksinen, Alan Lyles

**Affiliations:** a Clinical Pharmacy Group, Division of Pharmacology and Pharmacotherapy, Faculty of Pharmacy, University of Helsinki, PL 56, Viikinkaari 5, 00014 Helsinki, Finland; b School of Health and Human Services, College of Public Affairs, University of Baltimore, Baltimore, USA

**Keywords:** Medicine shortages, Obligatory storage, Obligatory storing, Security of supply

## Abstract

**Background:**

Since 1984, Finland has used storage obligations for essential medicines, requiring manufacturers and importers to maintain storage of certain medicines. This study aimed to investigate whether this type of obligatory storage system for human medicines is effective in preventing and mitigating medicine shortages.

**Methods:**

This is a retrospective register study, utilizing open data from Finnish, Norwegian, and Swedish national medicines authorities. We compared the proportion and median durations of shortages between medicines with and without storage obligations within Finland, and the proportion of shortages across Finland, Sweden, and Norway.

**Results:**

A total of 1910 shortage notifications met the inclusion criteria for the analysis of shortages within Finland. Medicines not subject to storage obligations experienced between 1.8 and 2.3 times more shortages during the study period compared to those covered by storage obligations. Additionally, the median duration of short-term shortages (lasting less than 21 days) was shorter for medicines that are subject to storage obligations.

The inter-country analysis included 1230 shortage notifications from Sweden, 1075 from Norway, and 1369 from Finland. In comparison to Finland, shortages of products equivalent to those subject to storage obligations were 2.5 times more common in Sweden, where no storage obligations are in place, and 2.4 times more common in Norway, where storage obligations are more limited.

**Conclusions:**

Our findings suggest that storage obligations for essential human medicines are associated with a lower frequency of shortages. This evidence provides support for other nations considering taking similar policy measures to reduce such shortages.

## Highlights

• Effectiveness was measured by both intracountry and intercountry comparison.
• Results were consistent in both comparisons.
• Finnish national storage obligation reduced national medicines shortages 45–56 %.
• Storage obligation reduced also median duration of shortages by 14–21.5 days.

## List of abbreviations

**Table:** 

ATC	Anatomical Therapeutic Chemical
Fimea	Finnish Medicines Agency
THL	National Institute for Health and Welfare (Finland)

## 1 Introduction

### 1.1 Medicine shortages threaten healthcare quality and safety

Medicine shortages are a rapidly increasing global threat,^1^, 2., 3., 4. leading some nations and international organisations to seek new strategies to prevent and better respond to shortages and global crises that threaten medicine supply chains.^5^, 6., 7. These strategies include improvements in monitoring shortages, increased production transparency, and requiring evidence of meeting regulatory obligations for storing surpluses of specified medicines.^8^^,^^9^

Some European countries, such as Finland and Switzerland, use obligatory storage to secure the availability of essential medicines.^2^ Many countries have adopted or are considering adopting such a system in the wake of the COVID-19 pandemic.^9^ “Just-in-time” management principles, where pharmaceutical manufacturers and pharmacies aim to maintain low inventory levels to minimize holding costs, are common in the industry but are a risk factor for medicine shortages.^10^, ^11^, ^12^ Thus, requiring minimums for medicine storage is a rational action to prevent medicine shortages. Overall, storing medicines has become a vital part of national resilience strategies.

In a 2024 study by Sabine Vogler,^9^ some form of obligatory storage system was reported for 20 countries out of 38 studied. In addition, five countries reported having some national stockpiles of medicines. Obligatory storage systems were also under discussion or preparation in several additional countries. However, in our search, we could find no earlier empirical studies on the effectiveness of national storage obligations in countering medicine shortages.

This study aims to investigate whether the evidence from the Finnish obligatory storage system for human medicines supports its effectiveness in preventing and mitigating medicine shortages.

### 1.2 The Finnish obligatory storage system

In Finland, a national obligation to store medicines has existed since 1984.^13^ The legislation aims to maintain a secure supply of medicines in normal circumstances and resilience during national or global emergencies.^14^ The obligation applies to Finnish pharmaceutical manufacturers, importers, public healthcare units (e.g., hospitals), and the National Institute for Health and Welfare (THL).^15^ They are obligated to maintain supplies of certain medicines sorted into 14 therapeutic classes.

The required storage quantity varies between two weeks' and three-, six- or ten-months' average consumption or sales, depending on which class the medicine belongs to, and which party is obligated to store it (Table 1).16., 17., 18. The list of products subject to storage obligations is updated annually by the Finnish Medicines Agency (Fimea) and includes medicines stored by manufacturers and importers. In 2022, the list contained 1364 medicinal products.^19^ The parties required to maintain an obligatory storage can choose which package sizes are used to meet the obligation. Alternatively, manufacturers, importers, and public health care units may choose to store the active substance, excipients, additives, and packaging materials used in the manufacture of the medicinal product.^15^

**Table 1:** Medicines subject to storage obligations in Finland grouped by class and the quantity of the storage obligation, as determined by average consumption/sales during a given time period.16., 17., 18.

	Class	Manufacturer	Importer	Public health care units	THL
1	Antimicrobials	10 months	10 months	6 months	
2	Medications for fluid and electrolyte disorders and parenteral nutrition	10 months	10 months	2 weeks	
3	Medications for cardiovascular disease and diuretics	6 months	6 months	6 months	
4	Medications for metabolic and endocrine disorders	6 months	6 months	6 months	
5	Analgesics, antirheumatic medicines, and medications for fever	6 months	6 months	6 months	
6	Local anaesthesia and general anaesthesia medications	6 months	6 months	6 months	
7	Medications for poisoning and vaccines	6 months	6 months	6 months	
	Vaccines that are part of the national vaccination programme	6 months			6 months
8	Medications for respiratory diseases	3 months	3 months	3 months	
9	Medications for digestive diseases	3 months	3 months	3 months	
10	Psychiatric medications	3 months	3 months	3 months	
11	Medications for neurological disorders	3 months	3 months	3 months	
12	Medications for eye diseases	3 months	3 months	3 months	
13	Antithrombotic and haemostatic medications	3 months	3 months	3 months	
	Oncology medication⁎			3 months	
14	Veterinary medicines	3 months	3 months		

⁎ Oncology medication contains both oncology medications and medications used to treat side effects from cancer drugs, immunostimulants and immunosuppressants; THL: National Institute for Health and Welfare.

The quantity stored is determined by the average consumption or sales for each medicine during a specific period in the previous year.^15^ Parties required to maintain an obligatory storage must annually report these data to Fimea. Manufacturers and importers then receive a yearly reimbursement from the Finnish National Emergency Supply Agency for the capital represented by the storage.

**Fig. 1:**
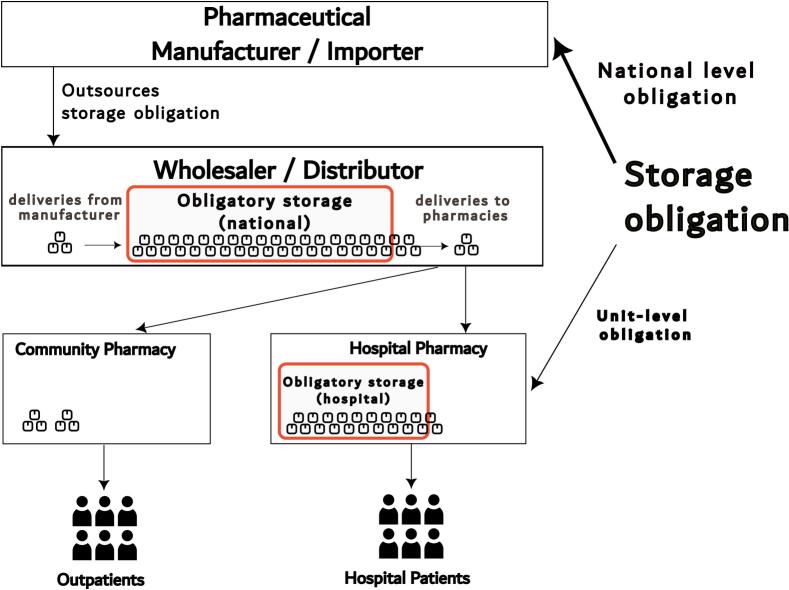
The Finnish medicines supply chain and obligatory storage.

Pharmaceutical distribution within Finland is organized through two major national wholesalers, although specific pharmaceutical products are usually distributed through just one of them.^20^ Many pharmaceutical manufacturers and importers have outsourced their responsibility for maintaining an obligatory storage to the wholesaler of their choice. Thus, obligatory storage is typically incorporated into a wholesaler's regular inventory, meaning that the material(s) in storage circulate with sales, minimizing the risk of having medicines expire while in an obligatory storage period (Fig. 1). Similarly, healthcare units' obligatory storage is commonly incorporated into the hospital pharmacy's regular inventory.^21^

Manufacturers, importers, and THL may apply to Fimea for a permit to maintain lower storage levels if a stored medicine experiences a shortage or if what is in inventory is about to expire during the storage period.^15^ This way, the released medicines can be used to prevent or mitigate shortages. Permits can be granted before or during a shortage. In 2022, only seven applications were denied out of a total of 324 such applications (2.2 %).^22^ Public healthcare units (hospitals) are exempt from applying for a permit to maintain lower storage levels during a shortage. Additionally, healthcare units are obligated to store medicines only for their own use, in contrast to manufacturers and importers, whose obligatory storage secures the supply of medicines at the national level.

## 2 Methods

### 2.1 Study design

**Fig. 2:**
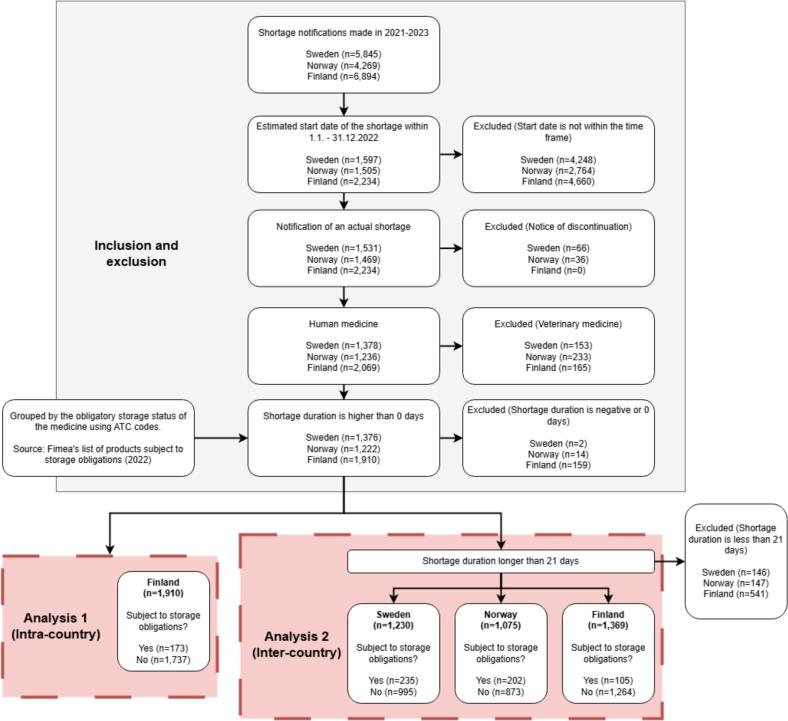
Visualization of the study design. ATC: Anatomical Therapeutic Chemical; Fimea: Finnish Medicines Agency.

Our retrospective register study utilised the Finnish, Swedish, and Norwegian shortage notification registers. The study material was augmented with information from the Finnish national medicine register and Fimea's list of products subject to storage obligations (Fig. 2).

Analyses were conducted in two ways: (1) by comparing the proportion and duration of shortages between medicines subject to storage obligations and those that are not within Finland, and (2) by comparing the proportion of shortages lasting more than 21 days across Finland, Sweden, and Norway.

Sweden and Norway were chosen as the reference countries as their pharmaceutical markets have many similarities to Finland, and they are all part of the larger Nordic pharmaceutical market. However, Sweden and Norway have organized the pharmaceutical wholesalers' supply security differently to Finland. In 2022 (the time frame for the material in this study), pharmaceutical wholesalers in Norway were obligated to maintain a storage of certain medicines corresponding to two months' average consumption.^23^ This had many similarities to the Finnish system, but the list of stored medicines was shorter, with approximately 41 ATC (Anatomical Therapeutic Chemical) codes, and the size of the required storage was smaller.

### 2.2 Data sources

The study material consisted of all medicine shortage notifications received by the Finnish, Swedish, and Norwegian medicine authorities from January 2021 to December 2023. Swedish and Norwegian shortage notification data were publicly available on the websites of the respective national medicine authorities at the time of data collection.^24^^,^^25^ The Finnish data were requested from Fimea. The Finnish national medicine register was used for data on available package sizes and the total number of medicinal products on the national market.^26^

In all three countries, the marketing authorisation holder is required to inform the national medicine authority of anticipated shortages at least two months prior to the start of a shortage.^2^^,^^27^ The required information for shortage notifications differed slightly between the countries but all three shortage notification registers included (i) the date of notification, (ii) the name of the medicine, (iii) its strength, (iv) package size, (v) active substance(s), (vi) the marketing authorisation holder, (vii) the ATC code, and (viii) the Nordic Product Number.^28^ In addition, each shortage's estimated start and end dates were provided for each notification. In all three registers, shortage notifications were displayed for each package size separately from one another.

### 2.3 Inclusion and exclusion criteria

Medicine shortage notifications with an estimated start date between the 1st of January 2022 and the 31st of December 2022 were included in the study material (Table 2). Notifications with no estimated start or end date were excluded, along with notifications with clearly erroneous estimated dates (e.g., estimated end year of 9999). In the Finnish data multiple notifications had been created on the same day for some medicines, and the details of these notifications differed from one another. These notifications were excluded from the study, as the correct information could not be determined.

**Table 2:** Inclusion and exclusion criteria for shortage notifications.

	Inclusion criteria	Exclusion criteria
Date of notification	Notification submitted during 2021–2023.	
Estimated start date	Between the 1st of January 2022 and the 31st of December 2022, inclusive.	No date given.
Estimated end date		No date given.
Medicine	Notifications on human medicines.	Notifications on veterinary medicines.
Notification type		Notifications on the discontinuation of medicines from the national market.
Duration of the shortage		The duration is ≤0 days.
Duration of the shortage (for intra-country analysis only)		The duration is less than 21 days.

Veterinary medicine shortage notifications were excluded (*n* = 165 Finland, *n* = 153 Sweden, *n* = 233 Norway). The Swedish and Norwegian shortage data also included notifications concerning the discontinuation of medicines from the national market (*n* = 66 Sweden, *n* = 36 Norway). These were removed from the data since the Finnish data did not include notifications of discontinuation.

### 2.4 Analysis of Finnish shortage notifications

The analysis of Finnish shortages was carried out by comparing the median shortage durations and the product-to-shortage ratio between medicines subject to storage obligations and those that are not. The comparison was conducted across four categories to examine how different conditions might influence the outcomes. The four categories used in the analysis were: (1) all reported shortages, (2) shortages lasting at least 21 days, (3) shortages in which none of the product's package sizes was available, and (4) shortages lasting at least 21 days with no available package sizes.

The duration of medicine shortages was calculated using the estimated start and end dates. Finnish data included the original notification and any further update notifications for the same shortage separately from one another. Only one notification of the same shortage was included for each medicinal product (i.e., the same brand, strength, form, and package size). If several notifications had been made regarding the same shortage, the estimated start and end dates of the latest notification were used to determine the duration.

### 2.5 Comparison of shortages across Sweden, Norway, and Finland

According to the Finnish shortage notification guidelines, there is no minimum duration for a shortage that should be reported. In contrast, Sweden requires reporting only for shortages lasting longer than three weeks,^2^ and the Norwegian data included only those exceeding two weeks. Although both the Swedish and Norwegian datasets contained some notifications for shorter shortages, many such cases were likely unreported due to the different guidelines. Therefore, to ensure data consistency across the three countries, all shortages lasting less than 21 days (three weeks) were excluded from the inter-country analysis (*n* = 541 Finland, *n* = 146 Sweden, *n* = 147 Norway).

### 2.6 Obligatory storage status

To compare medicines that are subject to storage obligations with those that are not, Finnish shortage notifications were grouped by Fimea's list of products subject to storage obligations for the year 2022.^19^ This list does not include oncology medicines subject to storage obligations by public healthcare units, and vaccines stored by THL and the manufacturer (classes 7 and 13 in Table 1). For this reason, in this study, these medicines are not among the medicines that are subject to storage obligations.

Medicines in the Swedish and Norwegian shortage notification data were also classified based on whether they would be part of Finland's obligatory storage system. A medicine was classified as being subject to storage obligations if the ATC code given in the notification was found in Fimea's list of products subject to storage obligations. However, some medicines with a matching ATC code were classified manually in cases where the formulation was not subject to storage obligations (e.g., adrenaline autoinjectors).

### 2.7 Statistical analysis and research ethics

The data were analysed using IBM SPSS Statistics for Windows, version 28. Descriptive statistics were used to calculate the median estimated duration for shortages. Statistically significant differences in median shortage durations were tested using the Mann-Whitney *U* test. Chi-square test was used to test for statistically significant differences in product-to-shortage ratios and intercountry ratios. Differences were considered statistically significant with a *p*-value of ≤0.05.

The material for this study consisted of publicly available retrospective register data, meaning that no research permit or ethical review was needed. This study was performed in accordance with the Finnish principles of responsible conduct of research.^29^

## 3 Results

### 3.1 Analysis of Finnish shortage notifications

From the Finnish medicine register, we found 8538 licensed pharmaceutical products on the market during 2022. Of these, 1364 (16.0 %) were medicines subject to storage obligations (Table 3).

**Table 3:** Shortage notifications in Finland for human medicines that are subject to storage obligations and not subject to storage obligations.

	Subject to storage obligations	Not subject to storage obligations	Total	p-Value
Number of products on the market	1364	7174	8538	

Total number of shortage notifications	173	1737	1910	
Shortage Notifications/Number of Products	0.13	0.24		
Shortages per product - ratio	0.52	1		<0.0001^a^
Median duration	27	41		0.004^b^

Shortages lasting a minimum of 21 days	105	1264	1369	
Shortage Notifications/Number of Products	0.08	0.18		
Shortages per product - ratio	0.44	1		<0.0001^a^
Median duration	58	59.5		0.713^b^

Shortages with other package sizes not available	103	984	1087	
Shortage Notifications/Number of Products	0.08.	0.14		
Shortages per product - ratio	0.55	1		<0.0001^a^
Median duration	27	48.5		0.001^b^

Shortages lasting a minimum of 21 days, other package sizes not available	63	737	800	
Shortage Notifications/Number of Products	0.05	0.10		
Shortages per product - ratio	0.45	1		<0.0001^a^
Median duration	55	69		0.263^b^

a Chi square test.

b Mann-Whitney median test.

From the Finnish medicine shortage register, we found 1910 shortage notifications for human medicines with an estimated start date between January 1st and December 31st, 2022. Of these, 173 (9.1 %) were products subject to storage obligations.

When excluding shortages lasting for less than 21 days, there were 1369 shortage notifications, 105 (7.7 %) of which concerned products subject to storage obligations.

Out of 1910 shortage cases, in 1087 cases other package sizes of the product were not available. 103 (9.5 %) of these cases concerned products subject to storage obligations.

After excluding both shortages which lasted for under 21 days and shortages where other package sizes of a product were not available, we found 800 shortages; 63 shortages for products subject to storage obligations and 737 shortages for products not subject to storage obligations. (*p* < 0.00001, chi-square).

The incidence of shortages was significantly lower, and the median duration of short-term shortages (less than 21 days) was shorter among products subject to storage obligations. No significant difference in median durations was found in longer shortages.

The exclusion of shortages for medicines with other package sizes available had only a minor effect on the results. However, excluding shortages lasting less than 21 days made the results on incidence even more pronounced.

### 3.2 Comparison of shortages across Sweden, Norway, and Finland

There were 1376 total shortage notifications which met the inclusion and exclusion criteria in Sweden, 1222 in Norway, and 1910 in Finland. After the exclusion of shortages shorter than 21 days, the number of shortages was 1230 in Sweden, 1075 in Norway, and 1369 in Finland (Table 4).

**Table 4:** Shortage notifications in Sweden, Norway, and Finland for human medicines that are equivalent to those subject to storage obligations and not subject to storage obligations.

	Subject to storage obligations/ equivalent products	Not subject to storage obligations	Total	Subject to storage obligations/ equivalent proportion	Proportion ratio	p-value (Chi-Square test)
Sweden	235	995	1230	19.1 %	1	
Norway	202	873	1075	18.8 %	0.98^1^	0.889
Finland	105	1264	1369	7.7 %	0.40^1^	<0.0001

1 Reference = Sweden.

In Sweden, after excluding shortages lasting less than 21 days, 19.1 % (235/1230) of shortages affected products equivalent to those subject to storage obligations in Finland.

In Norway, after excluding shortages lasting less than 21 days, 18.8 % (202/1075) of shortages affected products equivalent to those subject to storage obligations in Finland.

After excluding shortages lasting less than 21 days, 7.7 % (105/1369) of the shortages in Finland affected products subject to storage obligations.

The smaller proportion of shortages in products subject to storage obligations in Finland than equivalent products in either Sweden or Norway suggests that Finland's obligatory storage system is associated with decreased shortages. In Sweden, shortages in these products were 2.5 times more common than in Finland, and in Norway, they were 2.4 times more common (*p* < 0.00001, chi-square).

## 4 Discussion

As previous research has reported,^10^^,^^11^ the use of a just-in-time inventory policy is an underlying reason for shortages. Small national-level stock levels make countries more vulnerable to supply disruptions. National obligatory storage or other forms of stockpiling are important resilience mechanisms for an uninterrupted supply of essential medicines.^4^^,^^30^

According to our results, Finland's obligatory storage system is an effective policy measure for mitigating and reducing the duration of shortages of essential medicines.

In a survey of the national competent authorities of all European Union member states, all respondents from countries that employ obligatory storage reported them to be at least somewhat effective as a measure to ensure the supply of medicines.^3^ Our results support this assessment by the European national authorities, although the actual effectiveness may vary depending on the implemented measures, the level of actual compliance, and the national context.

Responsible national storage obligations or a stockpiling program should have flexibility, and it should be used as a long-term preventive measure. Unlike hoarding, which is reactive and increases demand during a supply crisis, responsible storing should increase the supply of targeted medicines during a supply crisis by releasing stored products and/or easing storage demands, and subsequently replenish storages during periods of sufficient global supply.

There is a risk that the rapid implementation of an obligatory storage policy, especially in large countries, could create shortages in global markets by increasing demand by accumulation to achieve storage targets. To avoid this, an obligatory storage system could be implemented gradually.

Some experts and industry stakeholders have argued for a more free trade-based approach on alleviating shortages and have opposed national export restrictions and stock requirements as regulatory hindrances to the flexible allocation of medicine supplies.^31^^,^^32^ However, there is evidence that a market-driven international distribution of medicines is uneven even inside the European Union.^33^ Especially for smaller and lower-priced markets, free trade is unlikely to guarantee a secure supply of medicines. This is an instance in which a market failure to achieve equilibrium between supply and demand requires a regulatory solution.

Besides obligatory storage, numerous national initiatives and measures have been introduced to mitigate medicine shortages. Research on their effectiveness and cost(s) should be performed to provide an empirical evidence base to support the formulation of pharmaceutical policies to reduce medicine shortages.

### 4.1 The cost of an obligatory storage system

An obligatory storage system carries with it compensation for the manufacturers and importers. This compensation is based on the value of the stored pharmaceuticals and the central bank interest rate, though it compensates only for the capital costs of obligatory storage.

The annual direct cost of Finland's obligatory storage system between 2019 and 2023 has varied between 1.7 and 4.4 million euro, with a mean of 2.4 million euro,^34^ which is 0.06 % of Finland's annual pharmaceutical sales (3756 million euro in 2022).^35^

Some studies have asserted that this reimbursement does not cover the full cost of obligatory storage.^36^, 37., 38. There are also costs of administration and inspections to be considered, for both authorities and companies. As such, theoretically it could increase the prices of medicines in Finland. However, in the only available analysis, a storage obligation was found to have a decreasing effect on the prices of some included products.^39^

Just as obligatory storage is not without costs, neither are shortages. There is a growing body of evidence about the monetary, clinical, and humanistic cost of shortages.^40^, ^41^, ^42^, ^43^, 44., 45 Although estimates covering the entirety of these costs are so far unavailable, these costs will likely, at least for essential medicines, exceed the costs of obligatory storage.

### 4.2 Limitations

Products subject to storage obligations are not necessarily fully equal with other products in distribution by price, patent status, drug form, market size, or other factors that might affect the probability of a shortage. However, based on our earlier research,^33^ we think that these potential biases in inter-country comparisons are minor. Storage obligations include products from therapeutic groups both less and more likely to have a shortage.

Medicines subject to storage obligations are generally not new patented medicines but older generic ones. This is due to the time required to achieve its status as an essential medicine, and the slow updating of the list of products subject to storage obligations. Since older generic medicines are more likely to have shortages^46^, ^47^, ^48^, ^49^, this could bias our results in intra-country comparison on the conservative side.

Likewise, differences in Nordic pharmaceutical markets and shortage notifications not related to medicines subject to storage obligations might create some unknown biases in our comparison of Finland, Sweden, and Norway. To eliminate these biases, we used the proportion of shortages instead of absolute numbers. Although all uncertainties could not be fully eliminated, the consistency of results in intra-country and inter-country comparisons strengthens our confidence in the reliability of the results.

Finally, since data on shortages is dependent on the information provided by the marketing authorisation holder, under-reporting or inaccuracies in the notifications may affect the results. However, we did not find major underrepresentation or inconsistencies within the data.

### 4.3 Generalizability of results

The effectiveness of Finland's obligatory storage system is based on (1) effective control of storage levels, (2) compensation of capital costs for manufacturers and importers, and (3) limiting obligations to essential medicines. We estimate that compliance with the Finnish obligatory storage system is high, which can't be taken for granted when regulations are not in individual companies' interest.^50^

The feasibility and therefore the effectiveness of storage obligations might be more limited in some countries. Suitable infrastructure and co-operation between the government and the private sector are the keys to the success of an obligatory storage system. The applicability and effectiveness of storage obligations in different contexts (such as low- and middle-income countries) warrant further research.

### 4.4 Obligatory storage in hospitals

In the Finnish pharmaceutical distribution system, marketing authorisation holders notify authorities of impending national shortages when they are unable to supply. Another level of obligatory storage exists for hospital pharmacies to maintain medication availability for hospital patients (Fig. 1). Assessing the effect of obligatory storage legislation on hospital pharmacy storing practices is outside the scope of the present study. It might be assumed that, in addition to the studied effects, obligatory storage in hospitals has additional effect to secure the availability of medications for hospital patients. Further research focusing specifically on this policy and these products is needed.

## 5 Conclusions

Based on our findings, Finland's obligatory storage system effectively mitigates shortages of essential human medicines. This evidence provides support for other nations considering taking similar policy measures to reduce such shortages.

## Availability of data and materials

Original data is public and obtainable from Finnish Medicines Agency, Swedish Medical Products Agency, and Norwegian Medical Products Agency. The datasets analysed during the current study are available upon reasonable request to the corresponding author.

## CRediT authorship contribution statement

**Reko Ravela:** Writing – review & editing, Writing – original draft, Visualization, Validation, Methodology, Investigation, Formal analysis, Data curation, Conceptualization. **Timi Aaltonen:** Writing – review & editing, Writing – original draft, Visualization, Validation, Methodology, Investigation, Formal analysis, Data curation. **Marja Airaksinen:** Writing – review & editing, Supervision. **Alan Lyles:** Writing – review & editing, Supervision, Methodology.

## Funding sources

One of the authors (RR) has received grants for research from Epilepsiatutkimusssäätiö (Epilepsy Research Foundation, Finland) and Yliopiston Apteekki (The University Pharmacy, Finland). Otherwise this research did not receive any specific grant from funding agencies in the public, commercial, or not-for-profit sectors.

## Declaration of competing interest

The authors declare that they have no known competing financial interests or personal relationships that could have appeared to influence the work reported in this paper.
